# Risk Factors for Invasive *Cryptococcus neoformans* Diseases: A Case-Control Study

**DOI:** 10.1371/journal.pone.0119090

**Published:** 2015-03-06

**Authors:** Ying-Ying Lin, Stephanie Shiau, Chi-Tai Fang

**Affiliations:** 1 Institute of Epidemiology and Preventive Medicine, College of Public Health, National Taiwan University, Taipei, Taiwan; 2 Department of Epidemiology, Mailman School of Public Health, Columbia University, New York, NY, United States of America; 3 Department of Internal Medicine, National Taiwan University Hospital, Taipei, Taiwan; University of Minnesota, UNITED STATES

## Abstract

**Background:**

*Cryptococcus neoformans* is a ubiquitous environmental fungus that can cause life-threatening meningitis and fungemia, often in the presence of acquired immunodeficiency syndrome (AIDS), liver cirrhosis, diabetes mellitus, or other medical conditions. To distinguish risk factors from comorbidities, we performed a hospital-based, density-sampled, matched case-control study.

**Methods:**

All new-onset cryptococcal meningitis cases and cryptococcemia cases at a university hospital in Taiwan from 2002–2010 were retrospectively identified from the computerized inpatient registry and were included in this study. Controls were selected from those hospitalized patients not experiencing cryptococcal meningitis or cryptococcemia. Controls and cases were matched by admission date, age, and gender. Conditional logistic regression was used to analyze the risk factors.

**Results:**

A total of 101 patients with cryptococcal meningitis (266 controls) and 47 patients with cryptococcemia (188 controls), of whom 32 patients had both cryptococcal meningitis and cryptococcemia, were included in this study. Multivariate regression analysis showed that AIDS (adjusted odds ratio [aOR] = 181.4; p < 0.001), decompensated liver cirrhosis (aOR = 8.5; p = 0.008), and cell-mediated immunity (CMI)-suppressive regimens without calcineurin inhibitors (CAs) (aOR = 15.9; p < 0.001) were independent risk factors for cryptococcal meningitis. Moreover, AIDS (aOR = 216.3, p < 0.001), decompensated liver cirrhosis (aOR = 23.8; p < 0.001), CMI-suppressive regimens without CAs (aOR = 7.3; p = 0.034), and autoimmune diseases (aOR = 9.3; p = 0.038) were independent risk factors for developing cryptococcemia. On the other hand, diabetes mellitus and other medical conditions were not found to be risk factors for cryptococcal meningitis or cryptococcemia.

**Conclusions:**

The findings confirm AIDS, decompensated liver cirrhosis, CMI-suppressive regimens without CAs, and autoimmune diseases are risk factors for invasive *C*. *neoformans* diseases.

## Introduction

*Cryptococcus neoformans* is a pathogenic fungus that causes life-threatening meningitis, the first case of which was reported in 1905 [1]. *C*. *neoformans* is ubiquitous [2]. The Centers for Disease Control and Prevention (CDC) of the United States estimated that there are approximately one million new cryptococcal meningitis cases every year worldwide, with more than 70% of these cases occurring in sub-Saharan Africa [3]. *C*. *neoformans* infection is therefore an important global health concern [4].

Current understanding of the pathogenesis of invasive cryptococcal diseases is primarily based on clinical case series [5–11] and mouse experiments [12–17], which suggest that host conditions that impair cell-mediated immunity (CMI) (e.g., human immunodeficiency virus [HIV] infection with acquired immunodeficiency syndrome [AIDS], immunosuppressive therapy) play a critical role. Liver cirrhosis has also been reported as a condition that may increase the risk of cryptococcosis [18]. Other reported possible risk factors include diabetes mellitus, lymphoproliferative malignancy, hematological malignancy, cancer, autoimmune diseases, and lung diseases [19–21]. However, ascertainment of a condition as a risk factor for a disease requires either a cohort study (to compare the incidence of disease between those with the condition and those without) or a case-control study (to compare the proportion of the condition between those with the disease and those without), in order to distinguish true risk factors from comorbidities. To date, no cohort study or case-control study has been conducted to examine the risk factors of invasive *C*. *neoformans* diseases.

The first case of cryptococcal meningitis in Taiwan was diagnosed in 1957 [22]. Large clinical case series on cryptococcal meningitis [23,24], cryptococcemia [25] and cryptococcal diseases [26] reported high rates of HIV/AIDS, immunosuppressive therapy, decompensated liver cirrhosis, malignancy, diabetes mellitus, and kidney diseases among the case patients. To distinguish risk factors from comorbidities, we performed a hospital-based, retrospective, density-sampled, matched case-control study.

## Methods

### Study Setting

This study was conducted at National Taiwan University Hospital (Taipei, Taiwan), a university-affiliated medical center with a 2,200-bed capacity, which provides both primary and tertiary referral care in northern Taiwan.

### Study Design

This was a retrospective, density-sampled, case-control study. Patients with cryptococcal meningitis or cryptococcemia who were diagnosed and hospitalized during 2002–2010 (cases) were compared with those who were hospitalized during the same time period but did not have cryptococcal meningitis or cryptococcemia (controls). The cases and controls were matched according to admission date, age, and gender.

### Ethical Statement

Patient medical records were retrospectively reviewed to obtain information on diagnosis, sites of *C*. *neoformans* infection, and potential risk factors. All personal information was anonymized. The study procedures were reviewed and approved by National Taiwan University Hospital’s institutional review board (No. 201101083RC). The institutional review board approved the exemption of informed consent.

### Recruitment of Cases

All of the patients with the ICD-9 diagnostic code 117.5 (cryptococcal diseases) upon either admission or discharge from January 1, 2002 to December 31, 2010 were identified using a computerized registry of inpatients. The patients’ medical records and microbiological reports were reviewed. All of the new-onset cases of *C*. *neoformans* meningitis (cryptococcal meningitis, confirmed by cerebrospinal fluid [CSF] culture) or *C*. *neoformans* fungemia (cryptococcemia, confirmed by blood culture) were included in the present study.

### Selection of Controls

The controls were selected from inpatients who did not have cryptococcal meningitis or cryptococcemia; they were individually matched to each case by admission date, age (within a 5-year range), and gender at a 2:1 (cryptococcal meningitis) or a 4:1 (cryptococcemia) ratio. We first obtained the list of 600,782 admissions during the period from January 1, 2002 to December 31, 2010 using a computerized registry of inpatients. We then randomly selected controls for each case from the inpatients who met the matching criteria, using a random number generator.

### Data Collection

The medical records were systematically reviewed for information regarding age, gender, and the presence of underlying diseases or conditions before the onset of invasive cryptococcal diseases. We assumed an induction time (i.e., the minimum time needed for a causal factor to induce the disease) of one month. Only those conditions that had been present at least one month prior to the onset of the cryptococcal diseases were included in this study.

The data were collected using a standardized recording format, which included items regarding the presence or absence of the following conditions: AIDS, decompensated liver cirrhosis, uremia, cytotoxic chemotherapy, CMI-suppressive therapy, organ transplantation, diabetes mellitus, lymphoma, leukemia/myeloma, solid cancer, other malignancies (e.g., gastrointestinal stromal tumor [GIST], sarcoma, thymoma), autoimmune diseases, cardiovascular disease, chronic obstructive pulmonary disease, asthma, bronchiectasis, pneumoconiosis, and gastrointestinal diseases. All of the data were independently verified by a second researcher.

In addition, the medical records were reviewed for a history of close contact with pigeons before the onset of the invasive cryptococcal diseases, including raising pigeons or visiting pigeon farms, if this information was recorded.

### Definitions

AIDS was defined using the CDC’s 1993 revised case definition [27]. Liver cirrhosis was considered to be decompensated if the Child-Pugh score [28] was B or C. CMI-suppressive therapy was defined to include corticosteroids, azathioprine, mycophenolate mofetil, cyclosporine, tacrolimus and other immunosuppressive drugs that inhibit T-cell functions. CMI-suppressive therapy was further classified to stipulate whether a regimen was prescribed with or without calcineurin inhibitors (CAs) (cyclosporine or tacrolimus), which have direct in vitro antifungal activity against *C*. *neoformans* [29]. Diabetes mellitus was defined as having a fasting plasma glucose level ≥ 126 mg/dL, having a random plasma glucose level ≥ 200 mg/dL, or already receiving antidiabetic agents or insulin to control sugar [30]. Uremia was defined as end-stage renal disease with uremic symptoms or as receiving dialysis for more than three months. Autoimmune diseases included systemic lupus erythematosus, rheumatoid arthritis, dermatomyositis, and pemphigus, among others.

### Statistical Analyses

Statistical analyses were performed using SAS 9.2 (SAS Institute, Cary, North Carolina, USA). When two proportions were being compared, a chi-square test was used. Fisher’s exact test was used when any value in the cells of the contingency table was smaller than five. Between-group differences for continuous data were compared using Wilcoxon rank sum test. Conditional logistic regression was used to analyze the risk factors. The exact method was used. All variables with p < 0.10 in the univariate analysis were included in the maximum model and underwent stepwise selection during the multivariate analyses. All statistical tests were two-tailed, and p < 0.05 was considered statistically significant.

## Results

### Cases

**Fig 1:**
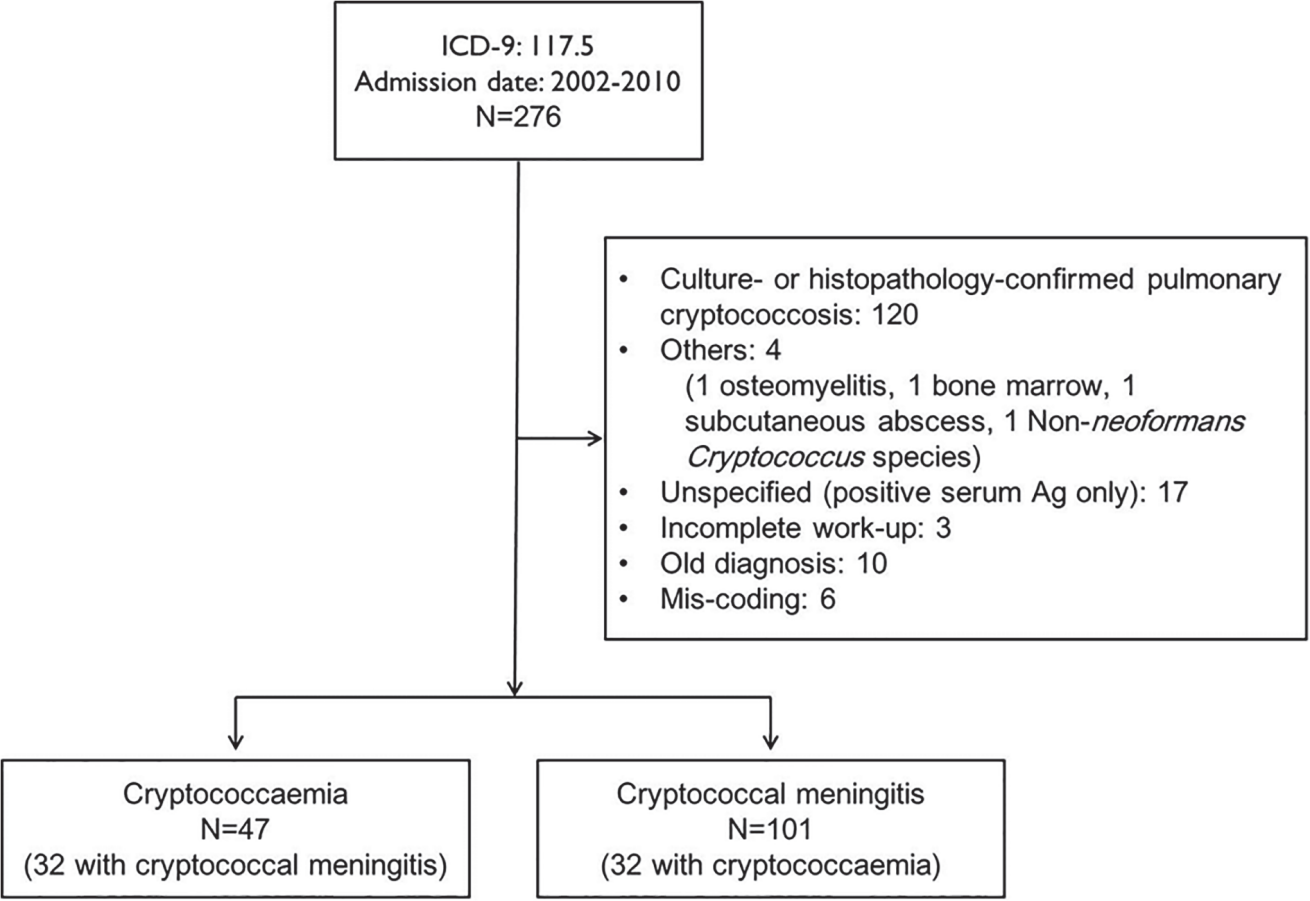
Flow chart of the recruitment of cases.

From January 1, 2002 through December 31, 2010, a total of 276 inpatients at National Taiwan University Hospital had the ICD-9 diagnostic code 117.5 upon admission or discharge. Of them, 116 had new-onset cryptococcal meningitis (n = 101) or new-onset cryptococcemia (n = 47), including 32 who had simultaneous cryptococcal meningitis and cryptococcemia. The remaining 160 patients did not meet the inclusion criteria, including those with pulmonary cryptococcosis (n = 120), other local involvement (n = 3), non-*Cryptococcus neoformans* fungemia (n = 1), an unspecified diagnosis (positive for serum cryptococcal antigen, but without a culture or histopathological confirmation; n = 17), an incomplete work-up (n = 3), an old diagnosis (n = 10), or a mis-coding (n = 6). Fig. 1 shows the flow chart of the recruitment of cases.

### Controls

A total of 326 controls were randomly selected from the 600,782 admissions during the period from January 1, 2002 through December 31, 2010, including 188 controls who were matched to the 47 patients with cryptococcemia (at a 4:1 ratio) and the 138 controls who were matched to the 69 patients who had cryptococcal meningitis but did not have cryptococcemia (at a 2:1 ratio).

### Epidemiologic Characteristics

Table 1 shows the characteristics of cryptococcal meningitis and cryptococcemia cases, including demographic features, underlying diseases, history of immunosuppressive therapy, and close contact with pigeons.

**Table 1:** Epidemiological characteristics of 101 *Cryptococcus neoformans* meningitis cases and 47 *C*. *neoformans* fungemia cases.

Characteristics	*C*. *neoformans* meningitis (N = 101)	*C*. *neoformans* fungemia (N = 47)	p-value
Age, range, years	20–86	24–79	
Age, mean ± SD, years	49.8 ± 17.2	51.6 ± 18.2	
Male	73 (72.3)	36 (76.6)	0.58
AIDS	42 (41.6)	21 (44.7)	0.72
Immunosuppressive therapy	24 (23.8)	13 (27.7)	0.68
CMI-suppressive therapy	23 (22.8)	13 (27.7)	0.52
Regimens without CAs	19 (18.8)	11 (23.4)	0.52
Prednisolone^a^	11	5	
Methylprednisolone^a^	1	1	
Prednisolone^a^ + azathioprine	4	3	
Prednisolone^a^ + chlorambucil	1	0	
Prednisolone^a^ + cyclophosphamide	1	1	
Prednisolone^a^ + rituximab + etoposide	1	0	
Dexamethasone^a^ + mephalan	0	1	
CA-based regimens	4 (4.0)	2 (4.3)	1.00
Tarcolimus + prednisolone^b^ + mycophenolate mofetil	3	1	
Cyclosporin + prednisolone^b^	1	0	
Cyclosporin + methylprednisolone^b^	0	1	
Cytotoxic chemotherapy	5 (5.0)	3 (6.4)	0.71
Cardiovascular disease	21 (20.8)	13 (27.7)	0.36
Autoimmune diseases	13 (12.9)	10 (21.3)	0.19
Decompensated liver cirrhosis	8 (7.9)	8 (17.0)	0.10
Diabetes mellitus	16 (15.8)	8 (17.0)	0.86
Gastrointestinal diseases	6 (5.9)	6 (12.8)	0.16
Lymphoma	4 (4.0)	3 (6.4)	0.68
Leukemia/myeloma	3 (3.0)	3 (6.4)	0.38
Cancer	14 (13.9)	6 (12.8)	1.00
Solid organ transplantation	4**†** (4.0)	1* (2.1)	1.00
Bone marrow/stem cell transplantation	0 (0)	0 (0)	-
Uremia	2 (2.0)	1 (2.1)	1.00
COPD	0 (0)	0 (0)	-
Asthma	0 (0)	0 (0)	-
Bronchiectasis	0 (0)	0 (0)	-
Pneumoconiosis	1 (1)	0 (0)	1.00
Close contact with pigeons	5 (5.0)	0 (0)	0.31

Data are no. (%) of patients, unless otherwise indicated. AIDS: acquired immunodeficiency syndrome; CAs: calcineurin inhibitors; CMI: cell-mediated immunity; COPD: chronic obstructive pulmonary disease.

†Heart (n = 2), kidney (n = 1), liver (n = 1).

*Heart (n = 1).

^a^ Mean dosage of steroid in CMI-suppressive regimens without CA: equivalent dose of prednisolone 20 mg/day in meningitis cases and 30 mg/day in cryptococcemia cases.

^b^ Mean dosage of steroid in CA-based regimens: equivalent dose of prednisolone 10 mg/day in meningitis cases and 55 mg/day in cryptococcemia cases.

The mean age of cryptococcal meningitis and cryptococcemia cases was approximately 50 years. Most cases (72.3%–76.6%) occurred in men. The age and gender distributions were similar between cases of cryptococcal meningitis and those of cryptococcemia (Table 1). The majority of patients younger than 40 years had AIDS, whereas the older age (≥50 years) group consisted primarily of non-AIDS patients.

AIDS (41.6%–44.7%) was the most common underlying condition in both the cryptococcal meningitis cases and the cryptococcemia cases, followed by immunosuppressive therapy (23.8%–27.7%), cardiovascular diseases (20.8%–27.7%), autoimmune diseases (12.9%–21.3%), decompensated liver cirrhosis (7.9%–17.0%), and diabetes mellitus (15.8%–17.0%). The pattern of underlying conditions was not significantly different between the cryptococcal meningitis cases and the cryptococcemia cases (Table 1).

In-hospital mortality was significantly higher in cryptococcemia cases compared with cryptococcal meningitis cases (23/47 [48.9%] vs. 28/101 [27.7%], p = 0.016). Of the 101 patients with cryptococcal meningitis, 32 (32%) had cryptococcemia, for whom in-hospital mortality occurred at double the rate of those who had cryptococcal meningitis but did not have cryptococcemia (14/32 [43.8%] vs. 14/69 [20.3%], p = 0.027). Cases with decompensated liver cirrhosis had a particularly high mortality, compared with cases without decompensated liver cirrhosis, in both cryptococcal meningitis group (6/8 [75.0%] vs. 22/93 [23.7%], p = 0.005) and cryptococcemia group (6/8 [75.0%] vs. 17/39 [43.6%], p = 0.137).

Table 2 shows the concomitantly involved sites in the 47 patients with cryptococcemia. Those with AIDS (n = 21) were more likely to have culture-proven meningitis than those with decompensated liver cirrhosis (n = 8) (19/21 [90%] vs. 3/8 [44.4%], p = 0.008). However, those patients with decompensated liver cirrhosis were significantly less likely than patients with AIDS to undergo a lumbar puncture for a CSF study (5/8 [62.5%] vs. 21/21 [100%], p = 0.023; Table 2).

**Table 2:** Characteristics of the 47 patients with cryptococcemia.

Involved sites†	All cases (N = 47) (%)	Decompensated cirrhosis (N = 8) (%)	AIDS (N = 21) (%)	p-value ξ (Decompensated cirrhosis vs. AIDS)
Meningitis	32 (68.1)	3 (37.5)	19 (90)	0.008
CSF culture (+)	31 (66.0)	3 (37.5)	19 (90)	-
CSF Cryptococcal Ag (+)	32 (68.1)	3 (37.5)	19 (90)	-
CSF culture (−)	5 (10.6)	2 (25.0)	2 (10)	0.568
CSF not checked	8 (17.0)	3 (37.5)	0 (0)	0.023
Peritonitis	3 (6.4)	3 (37.5)	1 (5)	0.076
Pleurisy	3 (6.4)	2 (25.0)	0 (0)	0.089
Lung (pathology-proven)	2 (4.3)	0 (0)	0 (0)	1.000
Urine	7 (14.9)	3 (37.5)	2 (10)	0.112
Bone and soft tissue	1 (2.1)	0 (0)	0 (0)	1.000
PTCD	1 (2.1)	1 (12.5)	0 (0)	0.310

PTCD: Percutaneous transhepatic cholangio-drainage.

ξ p-values were computed using Fisher’s exact test to compare cryptococcemia cases with decompensated liver cirrhosis and cases with AIDS.

†Culture-proven, unless otherwise indicated.

### Risk Factors for Cryptococcal Meningitis

The significant variables in the univariate analysis included AIDS, decompensated liver cirrhosis, CMI-suppressive regimens without CA, cytotoxic chemotherapy, cardiovascular disease, autoimmune diseases, cancer, and close contact with pigeons (Table 3). Diabetes mellitus was not a risk factor (crude odds ratio [OR] = 1.5; 95% CI: 0.7 to 3.2; p = 0.389). Multivariate regression analysis revealed the following three independent risk factors for cryptococcal meningitis: decompensated liver cirrhosis (adjusted OR = 8.5; 95% CI: 1.6 to 62.3; p = 0.008), AIDS (adjusted OR = 181.4; 95% CI: 28.2 to >999; p < 0.001), and CMI-suppressive regimens without CAs (adjusted OR = 15.9; 95% CI: 4.9 to 68.2; p < 0.001) (Table 4). Close contact with pigeons was a risk factor for cryptococcal meningitis in the univariate analysis (crude OR = 13.5; 95% CI: 1.8 to ∞; p = 0.008); however, this variable did not enter the final model.

**Table 3:** Univariate analyses of variables associated with cryptococcal meningitis.

Variables	Cases (N = 101)	Controls (N = 266)	Unadjusted OR (95% CI)	p-value
Decompensated liver cirrhosis	8	4	6.5 (1.5–39.4)	0.009
AIDS	42	3	118.8 (20.0–>999)	<0.001
Immunosuppressive therapy	24	57	-	-
CMI-suppressive therapy	23	15	-	-
Regimens without CAs#	19#	10#	5.5 (2.3–13.9)	<0.001
CA-based regimens	4	5	1.7 (0.3–8.1)	0.630
Cytotoxic chemotherapy	5	47	0.2 (0.1–0.7)	0.002
Solid organ transplantation	4**†**	5*	1.9 (0.4–8.8)	0.556
Bone marrow/stem cell transplantation	0	1	2.0 (0–78)	1.000
Diabetes mellitus	16	30	1.5 (0.7–3.2)	0.389
Cardiovascular disease	21	83	0.5 (0.2–0.9)	0.026
Uremia	2	4	1.2 (0.1–8.9)	1.000
Gastrointestinal diseases	6	24	0.9 (0.3–2.5)	1.000
Autoimmune diseases	13	8	4.6 (1.7–12.9)	0.001
COPD	0	5	0.6 (0–3.3)	0.655
Asthma	0	5	0.4 (0–2.1)	0.379
Bronchiectasis	0	2	1.7 (0–21.3)	1.000
Pneumoconiosis	1	0	2.0 (0.1–∞)	0.667
Lymphoma	4	5	2.2 (0.4–10.3)	0.422
Leukemia/myeloma	3	5	2.8 (0.5–15.6)	0.271
Cancer	14	85	0.3 (0.1–0.6)	<0.001
Others (GIST/sarcoma/thymoma)	0	5	0.5 (0–3.7)	0.546
Close contact with pigeons	5	0	13.5 (1.8–∞)	0.008

CMI: cell-mediated immunity; CAs: calcineurin inhibitors; COPD: chronic obstructive pulmonary disease; GIST: gastrointestinal stromal tumor.

†Heart (n = 2), kidney (n = 1), liver (n = 1).

* Heart (n = 2), kidney (n = 2), liver (n = 1).

# The mean dosage of steroid was significantly higher in cases received CMI-suppressive regimens without CA (n = 19) than that in controls received CMI-suppressive regimens without CA (n = 10) (equivalent dose of prednisolone: 20 vs. 5.4 mg/day, p = 0.041).

**Table 4:** Independent risk factors for cryptococcal meningitis.

Variables	Adjusted OR (95% CI)	p-value
AIDS	181.4 (28.2–>999)	<0.001
CMI-suppressive regimens without CAs	15.9 (4.9–68.2)	<0.001
Decompensated liver cirrhosis**†**	8.5 (1.6–62.3)	0.008

AIDS: acquired immunodeficiency syndrome. CMI: cell-mediated immunity; CAs: calcineurin inhibitors.

†Decompensated liver cirrhosis: Child-Pugh score of B or C.

### Risk Factors for Cryptococcemia

Univariate analyses identified AIDS, decompensated liver cirrhosis, CMI-suppressive regimens without CAs, autoimmune diseases, and cancer as the significant variables (Table 5). Diabetes mellitus was not a risk factor (crude OR = 1.4; 95% CI: 0.5 to 3.5; p = 0.650). Decompensated liver cirrhosis (adjusted OR = 23.8; 95% CI: 3.4 to 340.9; p < 0.001), AIDS (adjusted OR = 216.3; 95% CI: 24.2 to >999; p < 0.001), CMI-suppressive regimens without CAs (adjusted OR = 7.3; 95% CI: 1.1 to 57.5; p = 0.034), and autoimmune diseases (adjusted OR = 9.3; 95% CI: 1.1 to 135.7; p = 0.038) were independent risk factors for cryptococcemia (Table 6).

**Table 5:** Univariate analyses of variables associated with cryptococcemia.

Variables	Cases (N = 47)	Controls (N = 188)	Unadjusted OR (95% CI)	p-value
Decompensated liver cirrhosis	8	4	13.7 (2.7–134.6)	<0.001
AIDS	21	2	81.5 (13.1–>999)	<0.001
Immunosuppressive therapy	13	40	-	-
CMI-suppressive therapy	13	11	-	-
Regimens without CAs#	11#	9#	5.7 (2.0–17.6)	<0.001
CA-based regimens	2	2	4.0 (0.3–55.2)	0.362
Cytotoxic chemotherapy	3	31	0.3 (0.1–1.2)	0.106
Solid organ transplantation	1**†**	2*	2.0 (0.03–38.4)	0.980
Bone marrow/stem cell transplantation	0	0	-	-
Diabetes mellitus	8	25	1.4 (0.5–3.5)	0.650
Cardiovascular disease	13	69	0.6 (0.2–1.4)	0.252
Uremia	1	5	0.8 (0.02–8.4)	1.000
Gastrointestinal diseases	6	21	1.2 (0.3–3.6)	0.935
Autoimmune diseases	10	9	5.8 (1.9–19.9)	0.002
COPD	0	6	0.5 (0–2.6)	0.492
Asthma	0	5	0.6 (0–3.3)	0.655
Bronchiectasis	0	1	4.0 (0–76)	1.000
Pneumoconiosis	0	0	-	-
Lymphoma	3	5	2.6 (0.4–15.8)	0.394
Leukemia/myeloma	3	2	6.0 (0.7–71.8)	0.116
Cancer	6	61	0.3 (0.1–0.8)	0.008
Others (GIST/sarcoma/thymoma)	0	3	1.0 (0–6.9)	1.000

CMI: cell-mediated immunity; CAs: calcineurin inhibitors; COPD: chronic obstructive pulmonary disease; GIST: gastrointestinal stromal tumor.

†Heart (n = 1).

*kidney (n = 1), liver (n = 1).

# The mean dosage of steroid was significantly higher in cases CMI-suppressive regimens without CA (n = 11) than that in controls received CMI-suppressive regimens without CA (n = 9) (equivalent dose of prednisolone: 30 mg/day vs. 5 mg/day, p = 0.003).

**Table 6:** Independent risk factors for cryptococcemia.

Variables	Adjusted OR (95% CI)	p-value
AIDS	216.3 (24.2–>999)	<0.001
CMI-suppressive regimens without CAs	7.3 (1.1–57.5)	0.034
Autoimmune diseases	9.3 (1.1–135.7)	0.038
Decompensated liver cirrhosis**†**	23.8 (3.4–340.9)	<0.001

AIDS: acquired immunodeficiency syndrome. CMI: cell-mediated immunity. CAs: calcineurin inhibitors.

†Decompensated liver cirrhosis: Child-Pugh score of B or C.

## Discussion

To our knowledge, the present study is the first hospital-based, case-control study to investigate the risk factors for the occurrence of invasive *C*. *neoformans* diseases. The results showed that AIDS, decompensated liver cirrhosis, and CMI-suppressive regimens without CAs were three independent risk factors for cryptococcal meningitis and cryptococcemia in Taiwan. Moreover, autoimmune disease was an independent risk factor for cryptococcemia. On the other hand, diabetes mellitus and other medical conditions were not found to be risk factors of cryptococcal meningitis or cryptococcemia.

Both cryptococcal meningitis and cryptococcemia were included in the present study as invasive *C*. *neoformans* diseases cases. We had previously reported that cryptococcemia is a fulminant form of cryptococcal disease with a high acute mortality rate, especially for those with liver cirrhosis [25]. The present study further highlights that patients with cryptococcemia have an approximately two-fold higher mortality rate than patients who have had cryptococcal meningitis unaccompanied by cryptococcemia.

AIDS, the end stage of HIV infection [27], is characterized by profound impairment of CMI [5–11], which predisposes people to invasive *C*. *neoformans* diseases. Extrapulmonary cryptococcosis is an AIDS-defining illness [27]. Cryptococcal meningitis is one of the most common opportunistic infections in patients who develop AIDS [3,4]. The wide use of highly active antiretroviral therapy (HAART), which can help prevent the development of AIDS if initiated early in the course of HIV infection, led to a decline in the number of *C*. *neoformans* cases in North America and Western Europe [20,31]. Our study provides new epidemiologic evidence that confirmed that AIDS is an important risk factor for the occurrence of invasive *C*. *neoformans* diseases. Our results support the World Health Organization’s current advice on the prevention of cryptococcal diseases in HIV-infected patients, which emphasizes that the most important and cost-effective strategy is the early initiation of ART through expanded HIV testing and scaled-up access to HAART [4].

CMI has been shown to play a critical role in the host’s defense against *C*. *neoformans* in animal models [12–17]. Our study confirmed that CMI-suppressive therapy increases the risk of invasive *C*. *neoformans* diseases in humans. Of particular interest is the observation that only the regimen that did not contain a calcineurin inhibitor was found to be a significant risk factor. The virulence of *C*. *neoformans* depends on fungal calcineurin [32]. Calcineurin inhibitors, such as cyclosporine and tacrolimus, have in vitro antifungal activity against *C*. *neoformans* [29,33] and may mitigate the increased risk of *C*. *neoformans* diseases under CMI-suppressive therapy. However, because of the small numbers of subjects receiving cyclosporine- or tacrolimus-based CMI-suppressive regimens in the present study, whether such regimens actually did confer a lower risk of invasive *C*. *neoformans* diseases remains statistically inconclusive and needs to be clarified in future studies. It is advisable that all patients receiving CMI-suppressive therapy, regardless of their regimens, be educated to avoid exposure to environmental sources of *C*. *neoformans*, such as pigeons and Eucalyptus trees. Physicians should be aware of the risk of invasive *C*. *neoformans* diseases in these patients. Early diagnosis and prompt antifungal treatment can be life-saving.

Patients with autoimmune diseases, such as systemic lupus erythematosus (SLE), have been demonstrated to have defects in the functions of phagocytes, humoral immunity, and cellular immunity [34]. In a study involving 150 SLE patients, the functional impairment of T helper cells was found to be correlated with disease activity rather than the medication doses [34], which indicates that autoimmune disease per se is an independent factor that contributes to decreased CMI. In the present study, we confirmed autoimmune disease is an independent risk factor for the occurrence of cryptococcemia.

In the present study, we confirmed that decompensated liver cirrhosis is an independent risk factor for the occurrence of invasive *C*. *neoformans* disease. The pathogenesis mechanism could involve multiple aspects. First, although *C*. *neoformans* typically enters the human body via inhalation through the respiratory system, people may become infected by ingesting contaminated food [35]. The presence of collateral circulation in decompensated liver cirrhosis allows for ingested *C*. *neoformans* to bypass the liver scavenger system and directly enter the circulatory system, thereby causing cryptococcemia and being further disseminated into the central nervous system. In addition, deficiencies in complement [36] and chemotaxis [37] as well as lymphocyte hyporesponsiveness [38] have been reported. The impaired innate and cell-mediated immunity in persons with decompensated liver cirrhosis not only contributes to an increased risk for invasive cryptococcal disease, but also a high risk of mortality once the disease occurs [25]. The high mortality of patients with decompensated liver cirrhosis in the present study (6/8 [75%] in cryptococcal meningitis cases and 6/8 [75%] in cryptococcemia cases) is consistent with that reported by Jean et al. (9/11 [82%]) [25] and Singh et al. (26/32 [81%]) [18]. Whether early recognition and prompt antifungal therapy can reduce this high mortality remains to be determined.

Diabetes mellitus was present in 8.5% to 33% of cryptococcosis cases in reported series [21,23,26,39] and in 15.8% of cryptococcal meningitis cases and 17.0% of cryptococcemia cases in the present study. Nevertheless, diabetes mellitus is a relative common condition in population, and the prevalence increases with age (7.56% among Taiwanese men aged 40–59, 19.97% among Taiwanese men aged 60–79, in Year 2009) [40]. This highlights the necessity of using an age-matched control group in evaluating the role of diabetes. The lack of association between diabetes mellitus and cryptococcosis in the present case-control study implies that diabetes mellitus was a comorbidity in those with two diseases, and that diabetic patients are actually not at increased risk of invasive cryptococcal diseases.

The strengths of the present study include the use of a computerized inpatient registry and ICD-9 diagnostic codes to identify all cases of invasive *C*. *neoformans* diseases during the study period and the verification of the diagnoses through review of medical records and microbiologic reports. In addition, a standard data format was used to systematically collect information on potential risk factors from medical records. This method circumvents patient recall bias, which is commonly encountered in case-control studies.

Our study has several limitations. First, because the medical records may not have included comprehensive information on casual exposure to pigeons or Eucalyptus trees, we were unable to confirm that such exposure is a risk factor for invasive *C*. *neoformans* disease. Second, close contact with pigeons (including raising pigeons or visiting pigeon farms; n = 5) likely is an important factor in acquiring *C*. *neoformans* disease, and it was shown to be a risk factor in the univariate analysis (OR = 13.5, p = 0.008). However, the small number of cases limits the statistical power of the multivariate analysis to implicate close contact with pigeons as an independent risk factor.

In conclusion, AIDS, decompensated liver cirrhosis, CMI-suppressive regimens without CAs, and autoimmune diseases are four independent risk factors for the occurrence of invasive *C*. *neoformans* diseases, while diabetes mellitus and other medical conditions are not. The present study confirms previous clinical observations and animal studies on the critical role of impaired cell-mediated immunity in the pathogenesis of invasive *C*. *neoformans* diseases, and provides a more precise characterization of high-risk individuals.

## Supporting Information

S1 Dataset Cryptococcal meningitis and cryptococcemia dataset. (RAR)
